# Comparative modulation of the gut microbiota by date-derived products and refined sugar: a 16S rRNA gene taxonomic profile in healthy rats

**DOI:** 10.3389/fmicb.2026.1762458

**Published:** 2026-03-17

**Authors:** Randah M. Alqurashi, Muneera Q. Al-Mssallem, Ayesha W. Al-Majed, Mustafa Ibrahim Almaghasla, Sehad N. Alarifi

**Affiliations:** 1 Department of Food and Nutrition Sciences, College of Agriculture and Food Sciences, King Faisal University, Al-Ahsa, Saudi Arabia; 2 Plant Pests, and Diseases Unit, College of Agriculture and Food Sciences, King Faisal University, Al-Ahsa, Saudi Arabia; 3 Natural Science, Applied College - Shaqra, Shaqra University, Shaqraa, Saudi Arabia

**Keywords:** dates (*Phoenix dactylifera* L), dietary fiber, gut microbiota, natural sweeteners, prebiotics, rat model, table sugar

## Abstract

**Background:**

While refined dietary sugars are known drivers of microbial dysbiosis, natural date palm (*Phoenix dactylifera* L.) products contain complex matrices of fibers and polyphenols that may foster more favorable microbial environments. This study compared the effects of refined table sugar (TS) against various date-derived products—Date Sugar Powder (DSP), Dibs Cold-Treated (DCT), Dibs Heat-Treated (DHT), and Whole Date Fruit (WDF)—to assess their differential impact on the gut microbial community.

**Methods:**

Sprague–Dawley rats were assigned to five dietary intervention groups for 6 weeks. Gut microbial community structure and diversity were comprehensively assessed using 16S rRNA gene sequencing. Bioinformatic analyses were employed to identify taxonomic shifts and community-level differences across treatments.

**Results:**

16S rRNA gene sequencing analysis revealed that date-based treatments promoted microbial profiles that were distinct and compositionally more diverse than those induced by refined sugar. While the TS group consistently exhibited the lowest alpha diversity, beta diversity analysis (PERMANOVA, *p* = 0.001) confirmed significant community restructuring across groups, with WDF forming a unique cluster. Notably, the taxonomic response varied by processing method: DSP and DCT groups were characterized by an enrichment of *Parasutterella*, a genus previously associated in the literature with bile acid remodeling. The DHT group primarily promoted the abundance of *Prevotella* and *Alloprevotella*, taxa often recognized for their role in carbohydrate fermentation. Conversely, WDF fostered a unique enrichment of *Anaerovibrio* and the butyrate-associated genus *Roseburia*, suggesting that the whole-food matrix supports a distinct microbial niche.

**Conclusion:**

Natural date-derived products exert profound, product-specific modulatory effects on the gut microbiota, representing a compositionally superior alternative to refined sugar. Specifically, DCT showed a broad enrichment of health-associated taxa, while WDF promoted a unique assemblage of fiber-associated microbes. These results highlight the potential of date products as functional dietary alternatives and ingredients for the food industry, though further functional studies are required to confirm the metabolic implications of these taxonomic shifts.

## Highlights

- **Dibs under Cold Treatment (DCT)** was associated with a distinct gut microbiota profile characterized by the enrichment of *Parasutterella* and *Prevotella*. These taxa are frequently identified in literature as being correlated with **cholesterol regulation** and the **production of short-chain fatty acids (SCFAs)**.
- **Whole Date Fruit (WDF)** consumption promoted a unique microbial signature including *Anaerovibrio*, *Selenomonadaceae*, and *Roseburia*. This shift likely reflects the influence of the whole-food fiber matrix, which may support lineages typically involved in **lipid metabolism** and **butyrate synthesis**.
- **Refined Table Sugar** resulted in the lowest microbial richness and diversity among the groups. These findings suggest a **comparatively dysbiotic effect**, contrasting with the more diverse microbial profiles observed following the intake of natural, date-derived sweeteners.

## 1 Introduction

The gut microbiota represents a highly integrated microbial network that exerts a profound influence on host physiology, extending well beyond digestion to encompass immune balance, metabolic regulation, and the maintenance of intestinal barrier function (Subhash et al., 2025; Paul et al., 2025). Through the fermentation of indigestible dietary substrates, intestinal microbes generate essential metabolites—most notably short-chain fatty acids (SCFAs)—that contribute to epithelial integrity, regulate inflammatory signaling, and support systemic energy homeostasis (Subhash et al., 2025). Consequently, the gut microbiota serves as a central biological interface linking nutrition to health and disease, positioning it as a primary target for preventive and therapeutic nutritional strategies (Alhomsi and Kılıç Bayraktar, 2025; Paul et al., 2025).

Perturbations in this microbial ecosystem, commonly referred to as dysbiosis, have been increasingly implicated in the pathogenesis of numerous chronic conditions. Disruptions in microbial balance are associated with metabolic syndrome, obesity, type 2 diabetes, gastrointestinal disorders, and immune-mediated diseases, often accompanied by reduced microbial diversity and an enrichment of pro-inflammatory taxa (Grasa-Ciria et al., 2025; Yoo et al., 2024; Perler et al., 2023; Fu et al., 2022).

Diet remains the most influential and modifiable factor shaping gut microbial composition and function. Accumulating evidence indicates that diet plays a decisive role in this process, particularly through the intake of prebiotic polysaccharides that selectively stimulate beneficial microorganisms while limiting the expansion of pathogenic species (Alhomsi and Kılıç Bayraktar, 2025). These dietary inputs exert rapid and profound effects; short-term shifts can alter microbial diversity within days, while long-term patterns establish stable community structures (Ross et al., 2024). By providing substrates that reach the colon, diet facilitates the production of bioactive compounds—including SCFAs, secondary bile acids, and other metabolites—that significantly influence host physiology (Enriquez et al., 2023; Visekruna and Luu, 2021).

Different dietary components exert distinct effects on this landscape. Diets high in animal fat and protein are often associated with an increased abundance of bile-tolerant bacteria, such as *Bilophila* and *Alistipes*, which may contribute to systemic inflammation (Rinninella et al., 2023; Zhao et al., 2022; Jaagura et al., 2021). In contrast, plant-based diets rich in complex carbohydrates, fibers, and phytochemicals are linked to greater microbial diversity and the enrichment of beneficial taxa such as *Bifidobacterium* and *Lactobacillus* (Munteanu and Schwartz, 2024; Santhiravel et al., 2022). Dietary fibers, in particular, act as prebiotics by serving as fermentable substrates that stimulate the production of acetate, propionate, and butyrate, which support colonic health and improve metabolic outcomes (Facchin et al., 2024; Ashaolu et al., 2021; Holscher, 2017).

Dates (*Phoenix dactylifera L*.) are an important staple fruit consumed in various forms, including whole fruit, sugar powder, and date syrup (dibs). These products differ significantly in their processing methods, which may affect the retention of bioactive compounds and, in turn, their impact on gut health. For instance, heat processing can degrade certain phytochemicals, whereas cold processing may better preserve the fruit’s nutritional integrity and prebiotic potential (Alqurashi et al., 2025; Al-Hilphy et al., 2023; Alhamdan et al., 2021). Despite widespread consumption of these products, limited information is available on their comparative effects on the gut microbiota, particularly compared with refined table sugar. Therefore, this study aimed to investigate the impact of whole date fruit and various date-derived products (date sugar powder, heat-treated dibs, and cold-treated dibs) compared with refined table sugar on the gut microbiota composition and diversity in a healthy animal model.

## 2 Materials and methods

### 2.1 Samples preparation

Date whole fruit (WDF) (in the Tamer stage) and traditionally produced dibs of the Khalas variety were obtained from a private farm in Al-Ahsa, Saudi Arabia. Date sugar powder (DSP) of the Sukkari variety was sourced from Al-Amin Dates Factory, Al-Ahsa, Saudi Arabia. Table sugar (TS) (100% sucrose, Al-Osra brand, locally packaged) was obtained from Panda Markets, Al-Ahsa, Saudi Arabia. Dibs under cold treatment (DCT) were obtained from the cold-treated dibs, traditionally produced dibs of the Khalas variety were extracted from packed and pressed dates, with no further modifications. Dibs under heat treatment (DHT) was prepared according to the methodology in (Aleid and Haddadin, 2023) with minor modifications. Briefly, Khalas dates were mixed with distilled water in a ratio of 1:3 at 65 °C, with continuous mixing for 30 min. Next, the mixture was filtered through a muslin cloth, and the extracted liquid was concentrated to 74 °Bx at 70 °C by using Brix refractometer to obtain the final product.

### 2.2 Animals and diet

Thirty healthy male albino Sprague–Dawley rats (7–8 weeks old) were obtained from the College of Medicine animal house and fed laboratory chow and water ad libitum at King Faisal University, Al-Ahsa, Saudi Arabia. Animals were housed in standard polypropylene cages (six rats per cage) under controlled environmental conditions (22 ± 2 °C, 50–60% relative humidity, and a 12 h light/dark cycle and access to water and food. Following a one-week acclimatization period, rats were randomly assigned into five experimental groups (*n* = 6 per group) as following:

1. TS group – 50% table sugar + 50% basal diet
2. DSP group – 50% date sugar powder + 50% basal diet
3. DWF group – 50% date whole fruit + 50% basal diet
4. DHT group – 50% dibs under heat treatment + 50% basal diet
5. DCT group – 50% dibs under cold treatment + 50% basal diet

The basal diet was obtained from the local market (Al-Ahsa, Saudi Arabia), and the chemical composition of the basal diet was as follows: 25% protein, 60% carbohydrate, and 5% fat, 2.7% fiber, 1% vitamin mixture, 1% mineral mixture, 3.3% salt, and 2% chloride. Each group received its designated dietary treatment throughout the 6-week experimental period. At the start of the study, each group as provided with 100 g of its respective feed mixture per day, which was gradually increased to 150 g daily for 6 weeks (Table 1). Water was available at libitum. All animal handling procedures were performed in accordance with the institutional guidelines for the care and use of laboratory animals. Animal health and welfare were monitored daily; no clinical signs of metabolic distress or illness were observed during the study period. The experimental protocol was approved by the Research Ethics Committee of King Faisal University (Approval reference: KFU-REC-2024-MAY-ETHICS2304). Table 1 shows the nutritional composition of experimental diets.

**Table 1:** Nutritional composition of diet.

Experimental groups	Moisture %	Ash %	Fat %	Protein %	Fibers %	CHO %	Energy (cal)
TS	21.34 ± 0.26	2.28 ± 0.07	0.41 ± 0.01	1.75 ± 0.32	9.88 ± 0.25	64.45 ± 0.40	268.53 ± 0.28
WDF	20.26 ± 0.81	3.31 ± 0.03	0.48 ± 0.02	2.67 ± 0.30	12.88 ± 0.28	60.30 ± 1.84	256.21 ± 6.20
DSP	24.80 ± 0.64	3.10 ± 0.08	0.49 ± 0.01	3.47 ± 0.38	17.80 ± 0.60	50.57 ± 2.01	220.58 ± 6.51
DHT	22.24 ± 0.38	3.13 ± 0.14	0.45 ± 0.03	2.38 ± 0.35	10.35 ± 0.41	61.28 ± 0.70	258.69 ± 1.41
DCT	16.63 ± 0.76	3.35 ± 0.22	0.58 ± 0.02	2.69 ± 0.45	10.50 ± 0.21	66.48 ± 0.05	281.90 ± 1.58

TS, Table sugar; DSP, Date Sugar Powder; WDF, Date Whole Fruit; DCT, Dibs under Cold Treatment DHT, Dibs under Heat Treatment. Energy/100 g.

### 2.3 Samples collection

At the end of week 6, the rats were dissected. The cecal contents from all 30 rats were collected and immediately stored at −80 °C for DNA extraction and microbiota analysis.

### 2.4 Fecal microbiota analysis

#### 2.4.1 Deoxyribonucleic acid (DNA) fecal extraction

Genomic DNA was extracted from rat cecal contents using the Stool Genome DNA Extraction Kit (ELK Biotechnology, Denver, USA) following the manufacturer’s protocol. Briefly, ~180 mg of cecal content was lysed in Buffer SGE, incubated at 95 °C for 5 min, and clarified by centrifugation at 13,000 rpm at 4 °C for 10 min. The supernatant was treated with Solution InR and repeatedly centrifuged to remove impurities. DNA was then subjected to Proteinase K digestion, binding to a spin adsorption column, sequential ethanol-based washes, and elution in 50 μL Elution Buffer. Extracted DNA was stored at −80 °C until use (Elie et al., 2023).

DNA quality and integrity were assessed by 0.8% agarose gel electrophoresis in 0.5 × TBE buffer, and bands were visualized under UV illumination.

### 2.5 Library preparation and 16S rRNA gene sequencing

The V3–V4 hypervariable regions of the bacterial 16S rRNA gene were amplified using the universal primers 341F (5'-CCTACGGGNGGCWGCAG-3') and 805R (5'-GACTACHVGGGTATCTAATCC-3'). PCR amplification was performed using **Herculase II Fusion DNA Polymerase** (Agilent Technologies, USA) and the **Nextera XT Index V2 Kit** (Illumina, USA) following the **Illumina 16S Metagenomic Sequencing Library Preparation protocol** (Part # 15044223 Rev. B) (Illumina, Inc., 2022).

The PCR reaction system (25 μL) consisted of 2.5 μL of microbial genomic DNA (5 ng/μL), 5 μL of each forward and reverse primer (1 μM), and 12.5 μL of 2x KAPA HiFi HotStart ReadyMix. The thermal cycling conditions included an initial denaturation at 95 °C for 3 min, followed by 25 cycles of denaturation at 95 °C for 30 s, annealing at 55 °C for 30 s, and extension at 72 °C for 30 s, with a final extension at 72 °C for 5 min.

The quality of the resulting amplicons was verified via capillary electrophoresis. Sequencing was performed on the **Illumina platform** (Macrogen, Seoul, South Korea) using a paired-end 301 bp read length.

#### 2.5.1 Bioinformatics analysis and taxonomic inference

Bioinformatics processing of raw 16S rRNA sequences was conducted in R using the DADA2 pipeline, which facilitates high-resolution microbial profiling by modeling sequencing errors to distinguish true biological variation from technical noise (Callahan et al., 2016). This workflow enabled the inference of Amplicon Sequence Variants (ASVs), offering greater sensitivity and reproducibility than traditional OTU clustering (Callahan et al., 2017; Klindworth et al., 2013).

Initial quality control involved truncation of reads based on quality score decay and the removal of low-quality and PhiX sequences. Following dereplication and error modeling, paired-end reads were merged and chimeric sequences were identified and removed to ensure the integrity of the representative sequences. Taxonomic assignment was performed using a naive Bayesian classifier against the SILVA v138.1 database (Quast et al., 2013; Langille et al., 2013).

To facilitate downstream analysis, the resulting ASV feature table, taxonomic classifications, and metadata were integrated into a phyloseq object (McMurdie and Holmes, 2013; Le Chatelier et al., 2013). Robustness was further ensured by applying a quality filter to exclude low-prevalence ASVs and samples with insufficient sequencing depth, thereby focusing subsequent statistical interpretations on a highly representative dataset.

#### 2.5.2 Microbial diversity and abundance analysis

Microbial community structure was evaluated through alpha and beta diversity analyses. Alpha diversity (within-sample) was calculated using the Observed richness, Shannon, and Simpson indices, with significant differences tested via the Kruskal–Wallis test. Beta diversity (between-sample composition) was based on Bray–Curtis dissimilarity and visualized using Principal Coordinates Analysis (PCoA). Differences in community composition among groups were statistically determined using Permutational Multivariate Analysis of Variance (PERMANOVA), implemented with the vegan package (Oksanen et al., 2025). Differential Abundance Analysis was conducted using DESeq2 (Love et al., 2014), which utilizes a negative binomial generalized linear model to identify ASVs whose abundance was significantly altered across experimental groups. ASVs were deemed statistically significant at an adjusted *p*-value<0.05.

### 2.6 Statistical analysis and visualization

Data processing and statistical evaluations were executed in R (version 4.3.2) within the RStudio (2025.05.0+496) environment (Posit team, 2025). Data manipulation and structural wrangling were facilitated by the tidyverse suite (Wickham et al., 2019; R Core Team, 2023), ensuring a reproducible and streamlined workflow. For the characterization of community composition, the most abundant taxa within each treatment group were expressed as mean ± standard deviation (SD).

Differential abundance of specific microbial features was determined using Wald test statistics. A p-value of less than 0.05 (*p* < 0.05) was established as the threshold for statistical significance for all the results. All graphical representations, including taxonomic distributions and comparative visualizations, were generated using the ggplot2 package (Wickham, 2016).

## 3 Results

### 3.1 Impact of diet on body weight

**Figure 1:**
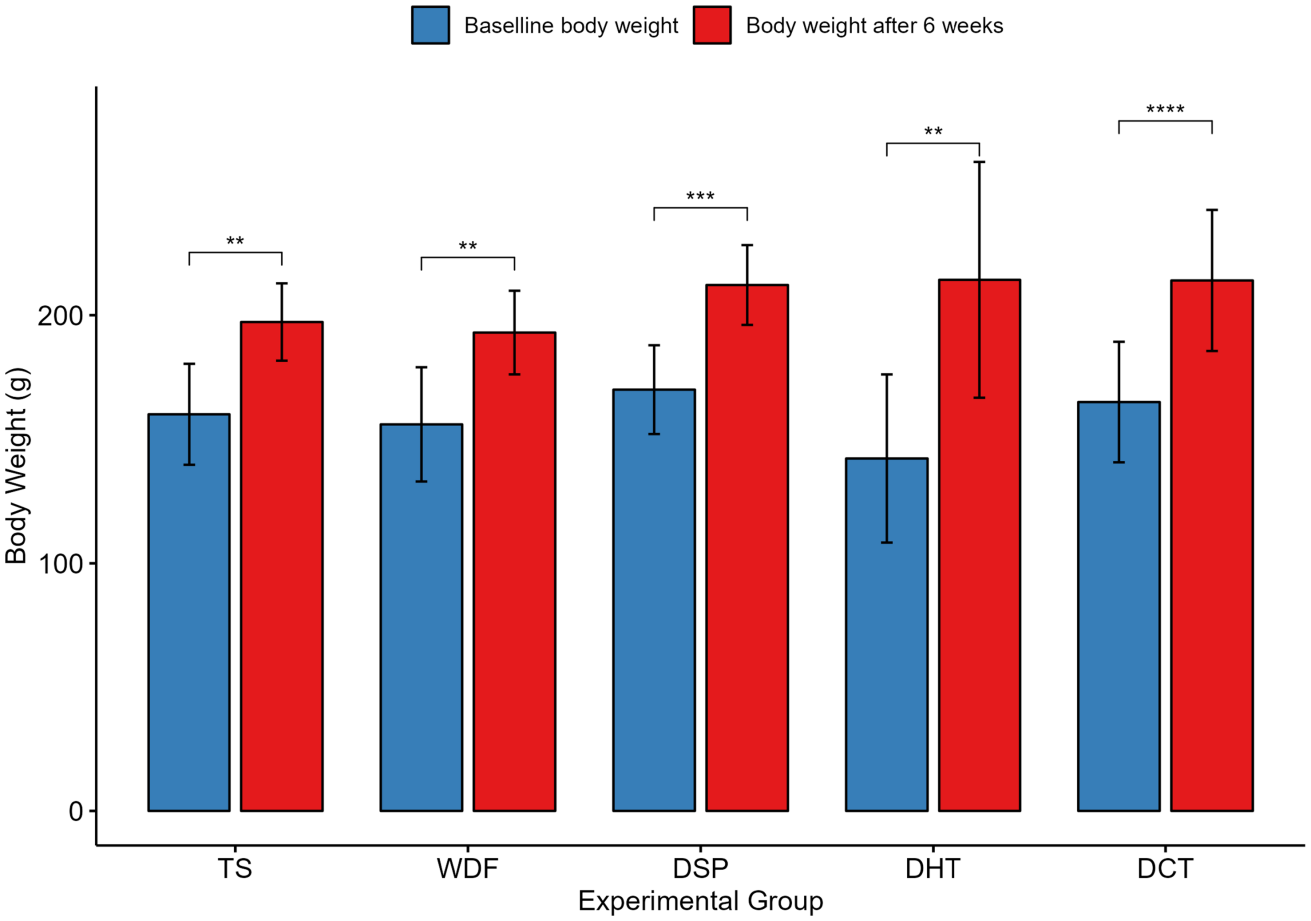
Body weight changes in rats after 6 weeks of treatment relative to baseline across different experimental groups: TS, Table sugar; DSP, Date Sugar Powder; WDF, Date Whole Fruit; DCT, Dibs under Cold Treatment; DHT, Dibs under Heat Treatment. Values are expressed as mean ± standard deviation. Asterisks indicate significant differences between baseline and week 6 within each group (*p < 0.05, **p < 0.01, ***p < 0.001, ****p < 0.0001; analyzed using a paired *t*-test).

To evaluate the metabolic impact of date-derived sweeteners versus refined sugar, body weight was monitored over a 6-week period (Figure 1). Although baseline weights were uniform across cohorts (144–172 g), all groups showed significant growth by the study’s conclusion (p < 0.05). Notably, the DHT group gained the most weight, approximately 55%, whereas the TS group showed the most conservative growth at 16%. Intermediate growth was observed in the WDF, DSP, and DCT groups (25–30%). Standard deviations remained relatively stable in the TS and DSP groups, whereas the DHT and WDF cohorts showed greater variability in individual weight-gain responses. This greater variability in the DHT and WDF cohorts further underscores that individual metabolic responses may fluctuate with the complexity and processing of the sugar source.

### 3.2 Diversity analysis

**Figure 2:**
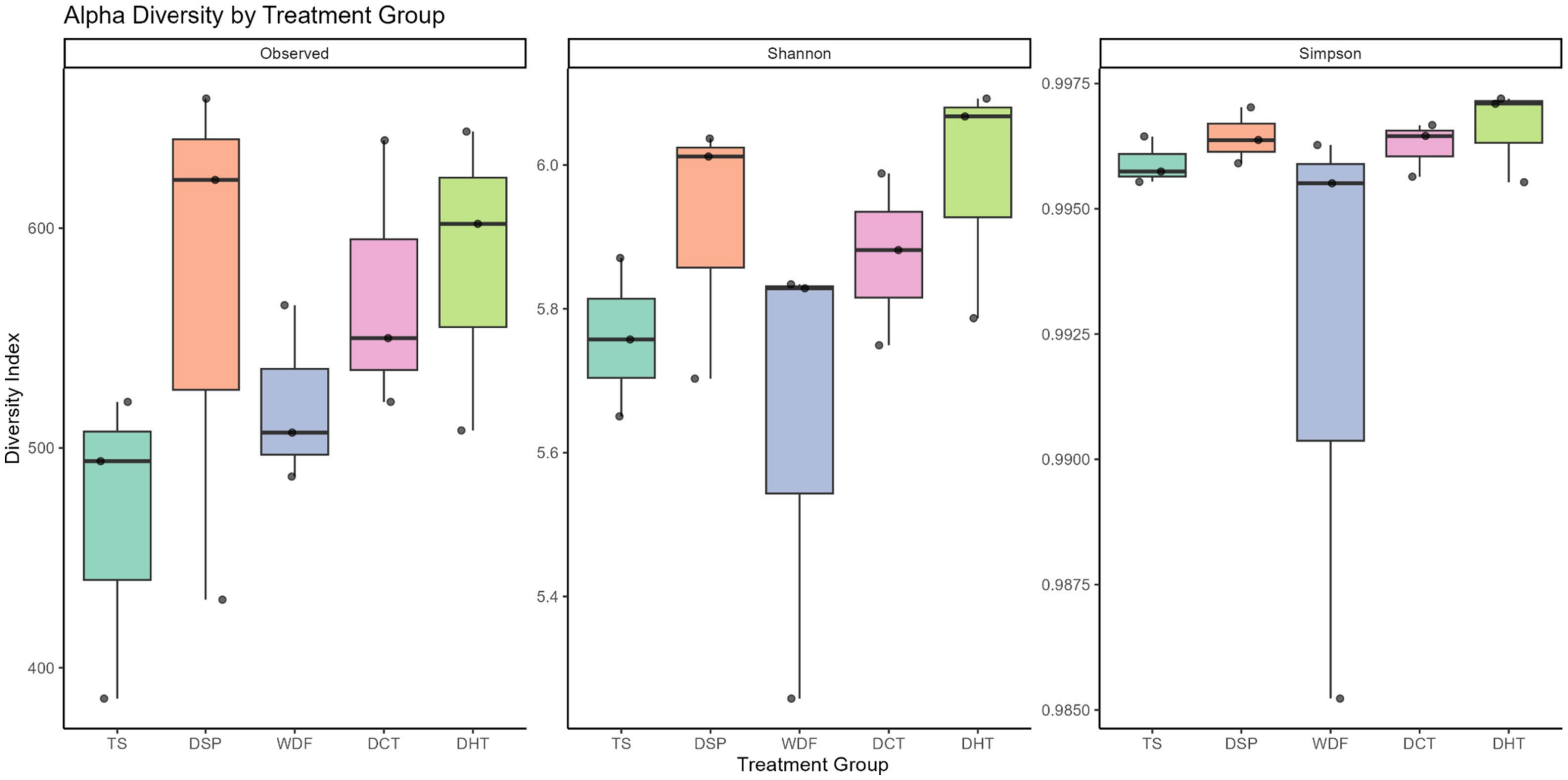
Alpha diversity metrics across treatment groups. Box plots illustrate the distribution of microbial richness and evenness using the Observed, Shannon, and Simpson indices for each of the five treatment groups. Statistical analysis via Kruskal–Wallis test indicated no significant differences in alpha diversity between the five treatment groups (*p* > 0.05). TS, Table sugar; DSP, Date Sugar Powder; WDF, Date Whole Fruit; DCT, Dibs under Cold Treatment DHT, Dibs under Heat Treatment.

An analysis of alpha diversity, which measures the richness and evenness of microbial communities within a sample, was conducted. The Observed richness showed that the DSP group had the highest median of 622, followed by DHT (602), DCT (550), WDF (507), and TS (494). For the Shannon diversity index, DHT had the highest median at 6.07, followed by DSP (6.01), DCT (5.88), WDF (5.83), and TS (5.76). The Simpson index values were consistently high and similar across all groups, with DHT at 0.997 and all other groups at 0.996. The Kruskal–Wallis test indicated that the observed differences in alpha diversity metrics were not statistically significant across the treatment groups. This lack of statistical significance may be due to the small sample size, with only three data points per treatment group. Figure 2 summarizes the alpha-diversity metrics across the groups.

**Figure 3:**
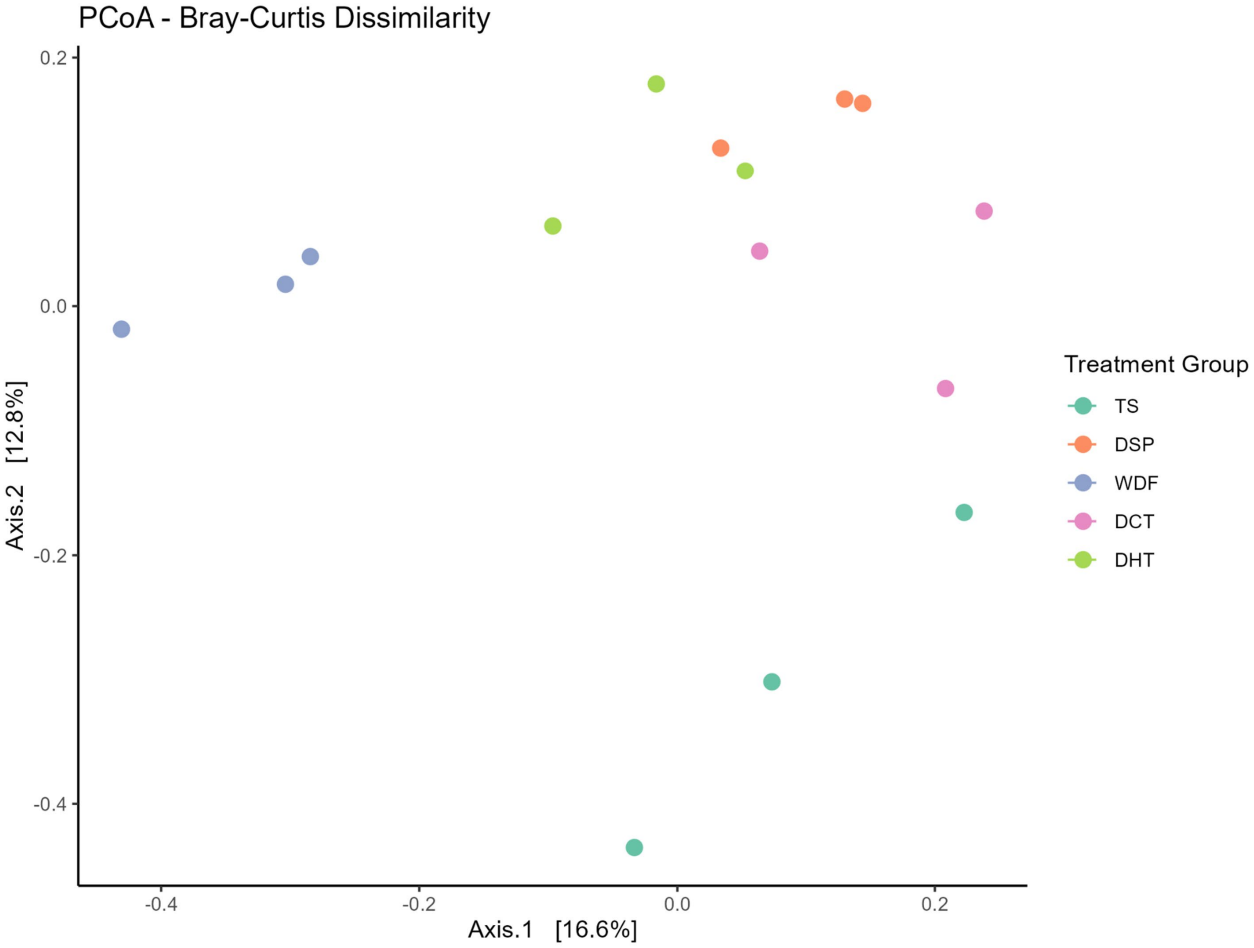
Principal Coordinates Analysis (PCoA) plot showing Bray–Curtis dissimilarity. The plot visualizes the beta diversity and clustering of microbial communities among the five treatment groups. The visual separation of the groups was confirmed by PERMANOVA (R^2^ = 0.4427, *p* = *p* = 0.001; see Table 4). TS, Table sugar; DSP, Date Sugar Powder; WDF, Date Whole Fruit; DCT, Dibs under Cold Treatment DHT, Dibs under Heat Treatment.

An analysis of beta-diversity, which measures the compositional differences between microbial communities, revealed a clear separation among the treatment groups. A Principal Coordinates Analysis (PCoA) plot, based on Bray–Curtis dissimilarity, showed that samples from the different groups clustered distinctly, suggesting that each treatment significantly shapes the overall microbial community structure. For example, the TS and WDF groups formed separate clusters from the DSP, DCT, and DHT groups, which themselves showed some degree of overlap. Figure 3 illustrates the PCoA analysis results.

This visual separation was confirmed by a Permutational Multivariate Analysis of Variance (PERMANOVA), which indicated a statistically significant difference in community composition across the groups (*p* = 0.001). The treatment factor explained 44.2% of the total variance (R^2^ = 0.4427) in the microbial community structure. This finding provides strong statistical evidence that different sugar sources and date fruit products significantly affect the overall microbial communities. PERMANOVA Results on Bray–Curtis Dissimilarity is shown in Table 2.

**Table 2:** PERMANOVA results on Bray–Curtis dissimilarity.

Factor	Degrees of freedom (Df)	Sum of squares (sum of sqs)	*R* ^2^	*F*-value	*p*-value
Treatment	4	1.52	0.44	1.99	**0.001**
Residual	10	1.91	0.56		
Total	14	3.44	1.00		

Bold values indicate statistical significance at *p* < 0.05.

### 3.3 Taxonomic profile

Based on the ASV assignments, the microbial communities were composed of 21 phyla, with Firmicutes (*n* = 2074), Bacteroidota (*n* = 1,609), and Proteobacteria (*n* = 196) being the most abundant. At the class level, the communities were primarily dominated by Clostridia (*n* = 1,688), Bacteroidia (*n* = 1,604), and Bacilli (*n* = 250). The most prevalent among 92 families included Muribaculaceae (*n* = 574), Lachnospiraceae (*n* = 476), and Prevotellaceae (*n* = 463). Finally, the most frequently observed genera were *Alloprevotella* (*n* = 90), *Rikenellaceae RC9 gut group* (*n* = 73), and *Bacteroides* (*n* = 66).

### 3.4 Relative abundance of dominant taxa

**Figure 4:**
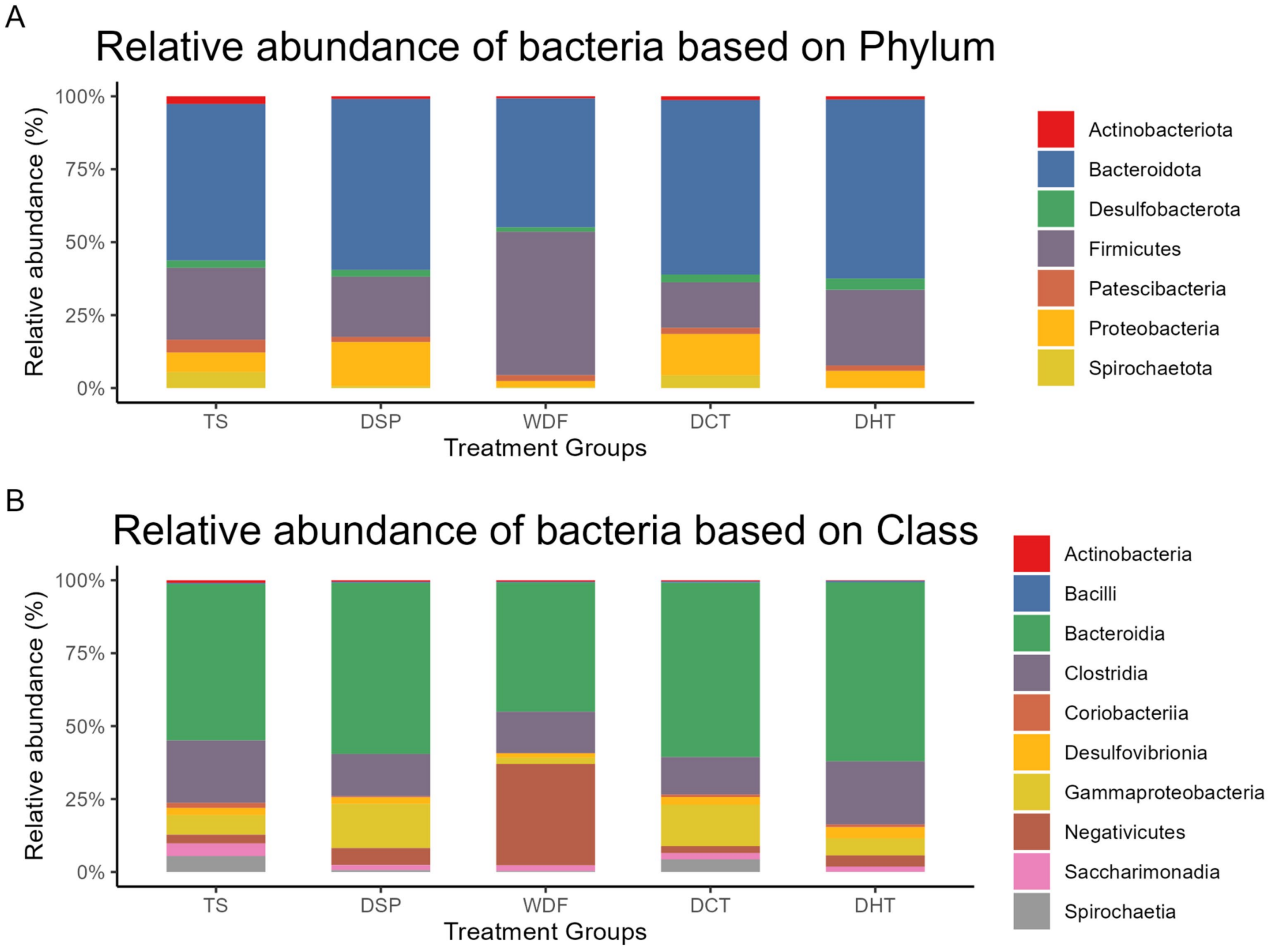
Relative abundance of dominant top 7 bacterial phyla **(A)** and top 10 classes **(B)**. This figure shows the compositional changes in microbial communities across the five treatment groups. TS, Table sugar; DSP, Date Sugar Powder; WDF, Date Whole Fruit; DCT, Dibs under Cold Treatment DHT, Dibs under Heat Treatment.

The relative abundance of dominant taxa was assessed at the phylum, class, family, and genus levels across the five treatment groups. At the phylum level, distinct patterns of abundance were observed. Actinobacteriota with an average total abundance of 1,088, was highest in the TS group and lowest in the WDF group. Bacteroidota had the highest average abundance of 48,411 among all groups and was distributed almost equally among them. Desulfobacterota (average abundance = 2,218) was highest in DHT and lowest in WDF. Firmicutes (average abundance of 23,577) were dominantly enriched in WDF, with the lowest abundance found in the DCT group. Patescibacteria were high in the TS group and lowest in DHT. Proteobacteria were selectively high in both DSP and DCT, while they were at their lowest in WDF. Lastly, Spirochaetota were selectively enriched in the DCT and TS groups. A similar pattern of differential abundance was observed at the class level. Bacteroidia were the most predominant class, with an equal distribution across all groups. Gammaproteobacteria were primarily enriched in DSP and DCT, with very low abundance in WDF. Negativicutes was selectively high in WDF, with low abundance in the DCT and TS groups. Spirochaetia were enriched in DCT and TS, with a negligible presence in WDF and DHT. The relative abundances of phyla and classes across the groups are depicted in Figure 4.

At the family level, distinct patterns of enrichment were observed across the treatment groups. The *[Eubacterium] coprostanoligenes* group was highly abundant in the DHT group but showed low enrichment in the DSP group. Atopobiaceae was present in high levels in the TS, DHT, and DCT groups, while Bacteroidaceae showed low abundance in the DSP group. Peptostreptococcaceae was dominant in the WDF and TS groups, with low levels in DSP. Prevotellaceae was a dominant family in the DHT, DSP, and DCT groups but was less abundant in TS. Selenomonadaceae was selectively enriched in WDF, with its lowest abundance in the TS group. Spirochaetaceae was predominantly present in DCT and TS, and Sutterellaceae was highly enriched in DSP and DCT, showing a negligible presence in WDF.

**Figure 5:**
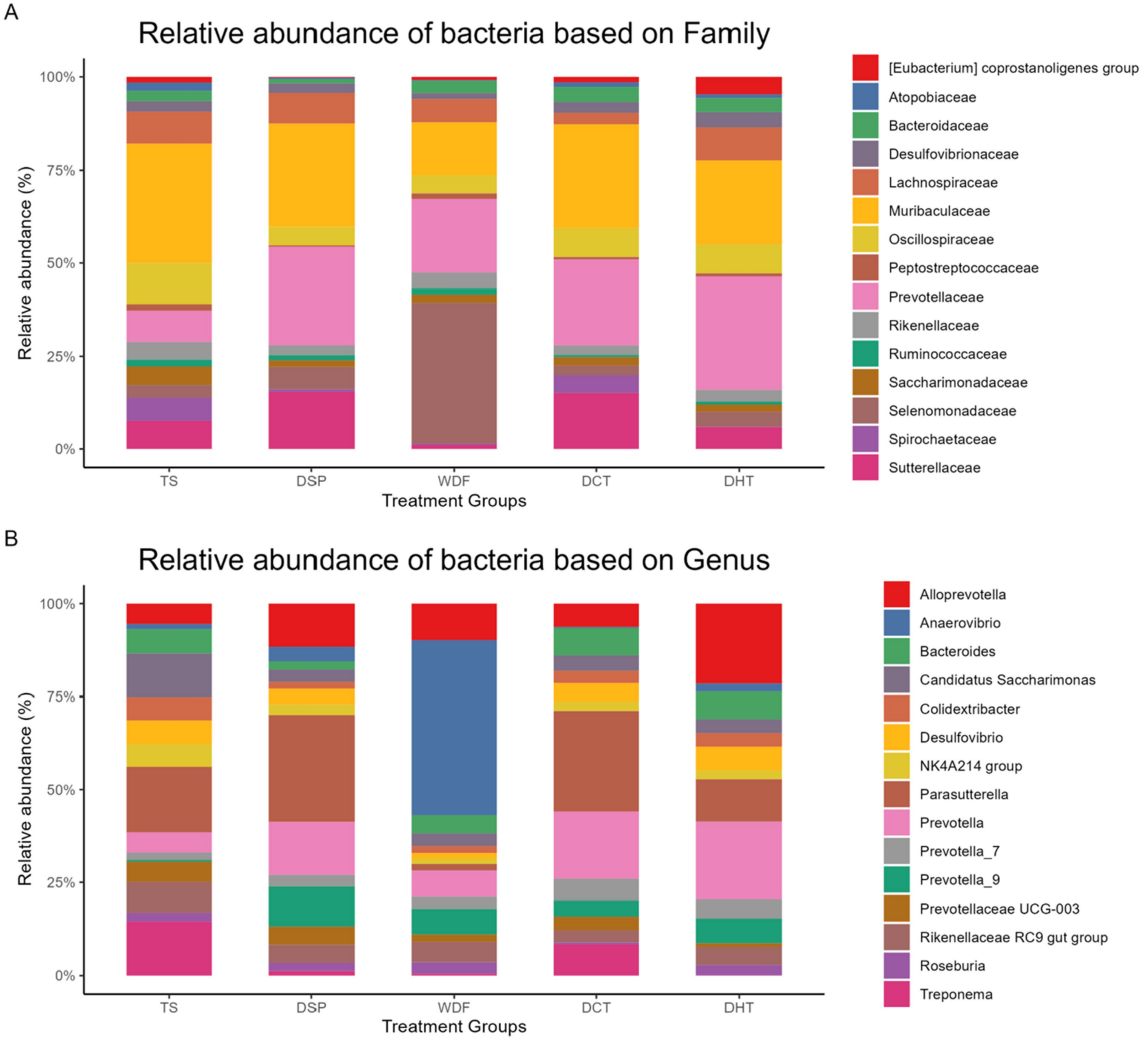
Relative abundance of dominant top 15 bacterial families **(A)** and top 15 genera **(B)**. This figure shows the compositional changes in microbial communities across the five treatment groups. TS, Table sugar; DSP, Date Sugar Powder; WDF, Date Whole Fruit; DCT, Dibs under Cold Treatment DHT, Dibs under Heat Treatment.

At the genus level, *Alloprevotella* was dominant in the DHT group, with its lowest levels in TS. *Anaerovibrio* was selectively high in WDF. *Parasutterella* was dominant in the DSP and DCT groups, while it was very low in WDF. Both *Prevotella* and Prevotella_7 were highly abundant in DHT and DCT, with very low presence in TS. Prevotella_9 was high in the DSP, WDF, and DHT groups, with a negligible presence in TS. *Prevotellaceae UCG-003* was very low in DHT. *Roseburia* was high in the WDF, DHT, and DSP groups but very low in DCT. Lastly, *Treponema* was selectively high in the TS and DSP groups, with negligible presence in WDF and DHT. The relative abundances of family and genus across the groups are depicted in Figure 5.

### 3.5 Top genera abundance per group

Table 3 presents the mean abundance and standard deviation of the top five most abundant genera within each of the five treatment groups, revealing distinct microbial community profiles with some shared genera across the groups. The TS group was predominantly enriched with *Candidatus Saccharimonas* (170 ± 145), *Lachnospiraceae* NK4A136 group (159 ± 157), and *[Eubacterium] siraeum* group (134 ± 90). Interestingly, *Treponema* was common between the TS group (182 ± 366) and the DCT group (195 ± 189). The DSP group showed a distinct enrichment of *Murimonas* (184 ± 319), *Prevotella_9* (136 ± 105), and *Parabacteroides* (136 ± 124). A shared abundance of *Parasutterella* was observed between the DSP (318 ± 329) and DCT (311 ± 240) groups, while *Prevotella* was common to the DSP (158 ± 126), DCT (208 ± 202), and DHT (225 ± 215) groups. The WDF group had a unique profile, with an exceptionally high enrichment of *Anaerovibrio* (896 ± 1,006). It also showed significant enrichment of *Romboutsia* (134 ± 148) and *Rikenellaceae* RC9 gut group (104 ± 53). *Alloprevotella* and *Roseburia* were shared between the WDF and DHT groups. The DCT group was also predominantly enriched with Bacteroides (120 ± 105) and shared Prevotella_7 with the DHT group (154 ± 140 for DCT; 126 ± 81 for DHT). Lastly, *Negativibacillus* was selectively enriched in the DHT group (123 ± 35).

**Table 3:** Mean abundance and standard deviation of the top five most abundant genera per treatment group.

Genus	TS^1^	DSP^1^	WDF^1^	DCT^1^	DHT^1^
*Treponema*	182 (366)	ND	ND	195 (189)	ND
*Candidatus Saccharimonas*	170 (145)	ND	ND	ND	ND
*Lachnospiraceae NK4A136 group*	159 (157)	ND	ND	ND	ND
*[Eubacterium] siraeum group*	134 (90)	ND	ND	ND	ND
*Coriobacteriaceae UCG-002*	134 (107)	ND	ND	ND	ND
*Parasutterella*	ND	318 (329)	ND	311 (240)	ND
*Prevotella*	ND	158 (126)	ND	208 (202)	225 (215)
*Prevotella_7*	ND	ND	ND	154 (140)	126 (81)
*Bacteroides*	ND	ND	ND	120 (105)	ND
*Alloprevotella*	ND	ND	100 (133)	ND	190 (225)
*Roseburia*	ND	ND	177 (91)	ND	138 (98)
*Negativibacillus*	ND	ND	ND	ND	123 (35)
*Murimonas*	ND	184 (319)	ND	ND	ND
*Prevotella_9*	ND	136 (105)	ND	ND	ND
*Parabacteroides*	ND	136 (124)	ND	ND	ND
*Anaerovibrio*	ND	ND	896 (1006)	ND	ND
*Romboutsia*	ND	ND	134 (148)	ND	ND
Rikenellaceae RC9 gut group	ND	ND	104 (53)	ND	ND

^1^Mean (SD). ND (Not detected), TS, Table sugar; DSP, Date Sugar Powder; WDF, Date Whole Fruit; DCT, Dibs under Cold Treatment DHT, Dibs under Heat Treatment.

### 3.6 Differential abundance of taxa

Differential abundance analysis was conducted using Wald test statistics, revealing 1,305 pairs with a statistically significant difference (*p* < 0.05). Notably, *Prevotella_9* had a lower abundance in the DCT group compared to DSP (*p* = 3.7 × 10^−17^), whereas *Oscillibacter* showed a higher prevalence in DCT (*p* = 1.0 × 10^−14^). The DSP group had more *Anaerovibrio* than DHT (*p* = 1.1 × 10^−16^), but less *Oscillibacter* (*p* = 2.9 × 10^−15^). Between WDF and DSP, the abundance of *Prevotella_7* increased in WDF (*p* = 7.0 × 10^−14^), while *Prevotellaceae UCG-001* decreased. The DCT group had higher *Anaerovibrio* than DHT (*p* = 2.3 × 10^−16^), but lower *Prevotella_9* (*p* = 1.8 × 10–15). The TS group had lower *Anaerovibrio* than WDF (*p* = 3.5 × 10^−7^) and higher *Parasutterella* (*p* = 4.6 × 10^−10^). Compared to DSP, the TS group had higher *Bacteroides* and *Desulfovibrio* (*p* = 8.6 × 10^−11^ and p = 1.0 × 10^−9^, respectively). However, *Desulfovibrio* was lower in TS than in both DCT (p = 2.9 × 10^−7^) and DHT (*p* = 2.8 × 10^−8^). Lastly, the WDF group had higher abundances of *Prevotella_9* and *Anaerovibrio* than the DCT group (*p* = 5.2 × 10^−16^ and p = 1.0 × 10^−15^, respectively). Additionally, WDF had more *Anaerovibrio* than DHT (p = 1.0 × 10^−20^) but less *Colidextribacter* (*p* = 4.1 × 10^−14^). Table 4 summarizes the top two pairs with the most significant differential abundance.

**Table 4:** Top two pairs with the most significant differential abundance based on the Wald test.

Group 1	Group 2	ASV ID	Genus	Status in group 2	*p*-value^1^	*p*_adj_ *^2^*
DSP	DCT	ASV0249	*Prevotella_9*	Decrease	1.2 × 10^−19^	3.7 × 10^−17^
DSP	DCT	ASV0609	*Oscillibacter*	Increase	1.1 × 10^−16^	1.0 × 10^−14^
DSP	DHT	ASV0020	*Anaerovibrio*	Decrease	1.7 × 10^−19^	1.1 × 10^−16^
DSP	DHT	ASV0609	*Oscillibacter*	Increase	3.7 × 10^−17^	2.9 × 10^−15^
DSP	WDF	ASV0242	*Prevotella_7*	Increase	1.3 × 10^−15^	7.0 × 10^−14^
DSP	WDF	ASV0386	*Prevotellaceae UCG-001*	Decrease	2.2 × 10^−15^	1.1 × 10^−13^
DCT	DHT	ASV0020	*Anaerovibrio*	Decrease	3.9 × 10^−19^	2.3 × 10^−16^
DCT	DHT	ASV0188	*Prevotella_9*	Increase	6.6 × 10^−18^	1.8 × 10^−15^
TS	WDF	ASV0020	*Anaerovibrio*	Increase	8.6 × 10^−9^	3.5 × 10^−7^
TS	WDF	ASV0239	*Parasutterella*	Decrease	3.3 × 10^−12^	4.6 × 10^−10^
TS	DSP	ASV0066	*Bacteroides*	Decrease	1.7 × 10^−13^	8.6 × 10^−11^
TS	DSP	ASV0112	*Desulfovibrio*	Decrease	3.9 × 10^−12^	1.0 × 10^−9^
TS	DCT	ASV0001	*Anaerovibrio*	Decrease	8.4 × 10^−13^	5.8 × 10^−10^
TS	DCT	ASV0074	*Desulfovibrio*	Increase	1.3 × 10^−9^	2.9 × 10^−7^
TS	DHT	ASV0074	*Desulfovibrio*	Increase	4.1 × 10^−11^	2.8 × 10^−8^
WDF	DCT	ASV0249	*Prevotella_9*	Decrease	9.0 × 10^−19^	5.2 × 10^−16^
WDF	DCT	ASV0001	*Anaerovibrio*	Decrease	3.5 × 10^−18^	1.0 × 10^−15^
WDF	DHT	ASV0020	*Anaerovibrio*	Decrease	1.8 × 10^−23^	1.0 × 10^−20^
WDF	DHT	ASV0355	*Colidextribacter*	Increase	3.6 × 10^−16^	4.1 × 10^−14^

^1^Wald test; ^2^Benjamini-Hochberg (BH) method. TS, Table sugar; DSP, Date Sugar Powder; WDF, Date Whole Fruit; DCT, Dibs under Cold Treatment DHT, Dibs under Heat Treatment.

## 4 Discussion

The therapeutic potential of dietary fibers and polysaccharides in managing metabolic disorders is increasingly attributed to their capacity to modulate gut microbiota composition (Subhash et al., 2025). In this study, we utilized 16S rRNA gene sequencing to characterize the microbial landscape of rats subjected to various dietary interventions.

By the conclusion of the six-week intervention with table sugar and date-based treatments, all experimental groups exhibited a consistent upward trend in body mass. These findings suggest that while all sugar sources support weight gain in this model, the physical form and processing method of date products—specifically heat-treated dibs—may influence the rate of weight gain differently than whole fruit or refined sucrose. Changes in body weight are closely related to how dietary fibers and polysaccharides affect gut bacteria (Cronin et al., 2021; Holscher, 2017). This means that how a sweetener is processed can have a greater effect on metabolism than its calorie content alone. For example, heat treatment can break down complex polysaccharides into simpler sugars, which may lead to stronger insulin responses and increased fat storage (Lu et al., 2025; Birlouez-Aragon et al., 2010). Understanding these specific mechanisms helps clarify how processing methods influence weight gain. In addition, the Whole Date Fruit (WDF) contains intact dietary fiber, which helps slow digestion. In contrast, the concentrated syrups (DHT and DCT) lack this fiber, leading to rapid calorie absorption (Dreher, 2018; Aljutaily et al., 2022; Giuntini et al., 2022). Therefore, while dates can be a natural alternative to table sugar, heat processing can significantly increase their potential to cause weight gain. Weight gain from high-dose supplementation is mainly a result of increased calorie intake, not a sign of health problems. The treatments added calories and also changed the gut bacteria in a positive way. We saw a rise in beneficial bacteria that produce Short-Chain Fatty Acids (SCFAs), especially *Roseburia*. This suggests that the gut is managing the extra calories well. *Roseburia* produces butyrate, which helps protect the intestine and improve insulin sensitivity. This means increased body fat is separate from the inflammation often linked to metabolic diseases (Pan et al., 2024; van Deuren et al., 2022; Fei and Zhao, 2013). Overall, this shows how beneficial gut bacteria can help offset the health risks of consuming more calories.

Our analysis revealed that date-based treatments promote a richer, more functionally diverse microbial profile than refined table sugar (TS), which consistently induced less favorable compositional shifts. Notably, the nature of these responses varied according to the degree of processing, underscoring the critical role of food matrix complexity in shaping gut ecosystem structure.

A striking finding in the WDF group was the elevated Firmicutes-to-Bacteroidota (F/B) ratio. While an increased F/B ratio has historically been implicated in models of obesity and dysbiosis (Magne et al., 2020), our results suggest a more nuanced interpretation. The enrichment of Firmicutes in the WDF group likely reflects a specialized adaptation to the whole fruit’s complex fiber matrix. Many members of the Firmicutes phylum are recognized in literature as primary butyrate producers; thus, an increase in their relative abundance may represent a shift toward a community with higher butyrogenic potential rather than a maladaptive state (Magne et al., 2020; Singh et al., 2023).

Furthermore, WDF fostered a unique community profile marked by an exceptional enrichment of *Anaerovibrio* and a high relative abundance of the family *Selenomonadaceae*. These taxa are often identified in studies involving lipid-rich or complex-carbohydrate diets, suggesting that the intact fiber and nutrient content of the whole fruit supports a distinct microbial niche (Alhomsi and Kılıç Bayraktar, 2025; Zhao et al., 2018). The simultaneous high abundance of *Roseburia*—a genus typically associated with intestinal health—further underscores WDF’s potential to support a beneficial microbial environment. This taxonomic assemblage is characteristic of ecosystems where primary fermenters and secondary degraders coexist, a structure often associated with stable SCFA production in other dietary models (Eid et al., 2014).

In contrast, processed products—Date Sugar Powder (DSP) and Dibs (DCT and DHT)—showed a high abundance of the genus *Parasutterella*. The interpretation of *Parasutterella* enrichment is complex, as this genus has been observed at elevated levels in diverse clinical conditions, including inflammatory bowel disease, obesity, and social anxiety disorder (Sun et al., 2024; Zhang et al., 2023; Santana et al., 2022). However, its role in the gut-metabolic axis appears to be highly context-dependent. Recent research indicates that *Parasutterella* is a core component of the human and murine gut microbiota that thrives on specific dietary carbohydrates without necessarily promoting inflammatory signaling (Henneke et al., 2022).

In the present study, enrichment of *Parasutterella* in the DCT group was accompanied by increases in well-recognized beneficial taxa, such as *Bifidobacterium* and *Prevotella*. This suggests that within the healthy rat model, *Parasutterella* may act as a specialized member of a consortia involved in the breakdown of date-derived carbohydrates or in bile acid remodeling (Ju et al., 2019; Bowman and Ducklow, 2015). The absence of a corresponding bloom in pro-inflammatory Proteobacteria across these groups further supports the hypothesis that this shift represents a metabolic adaptation to natural sweeteners rather than a state of dysbiosis (Shin et al., 2015).

The efficacy of DCT (Cold-Treated) compared to DHT (Heat-Treated) is particularly noteworthy. Cold processing likely preserves a higher concentration of bioactive polyphenols and thermosensitive polysaccharides that are otherwise degraded by heat (Alqurashi et al., 2025; Al-Hilphy et al., 2023; Alqahtani et al., 2023; Sharoba et al., 2022). These preserved compounds may act as selective substrates for a more diverse assemblage of health-associated microbes. For instance, the concurrent promotion of *Prevotella* in the DCT group is significant, as this genus is widely documented as an efficient degrader of complex dietary carbohydrates and a producer of beneficial SCFAs (Han et al., 2024; Betancur-Murillo et al., 2023; Miao et al., 2022).

Our broader community-level analyses support these specific taxonomic shifts. Beta diversity analysis revealed robust, statistically significant differences across treatments (PERMANOVA, *p* = 0.001), confirming that dietary inputs primarily drive a restructuring of the microbial community, even when overall alpha diversity remains stable. While alpha diversity metrics did not reach statistical significance—likely due to the pilot nature of the sample size—the TS group consistently exhibited the lowest richness and evenness. This trend aligns with established literature linking refined sugar consumption to reduced microbial diversity, a hallmark of gut dysbiosis (Dalamaga and Tsigalou, 2024; Garcia et al., 2022; Alasmar et al., 2023; Lu et al., 2025; Satokari, 2020).

Despite these significant findings, this study is primarily taxonomic in nature. While 16S rRNA sequencing provides a clear map of microbial community structure, it does not directly measure the functional metabolic output or active biochemical pathways. Future studies should integrate shotgun metagenomics and metabolomics—specifically targeting SCFAs and bile acids—to directly quantify the metabolic consequences of these observed taxonomic shifts. Further, future work would also include blood biochemistry to complement the microbial data.

## Data Availability

The datasets presented in this study can be found in online repositories. The names of the repository/repositories and accession number(s) can be found below: PRJNA1427961 and https://www.ncbi.nlm.nih.gov/sra/PRJNA1427961.
